# Thrombocytosis: Diagnostic Evaluation, Thrombotic Risk Stratification, and Risk-Based Management Strategies

**DOI:** 10.1155/2011/536062

**Published:** 2011-06-08

**Authors:** Jonathan S. Bleeker, William J. Hogan

**Affiliations:** Division of Hematology, Department of Medicine, Mayo Clinic, 200 First Street SW, Rochester, MN 55905, USA

## Abstract

Thrombocytosis is a commonly encountered clinical scenario, with a large proportion of cases discovered incidentally. The differential diagnosis for thrombocytosis is broad and the diagnostic process can be challenging. Thrombocytosis can be spurious, attributed to a reactive process or due to clonal disorder. This distinction is important as it carries implications for evaluation, prognosis, and treatment. Clonal thrombocytosis associated with the myeloproliferative neoplasms, especially essential thrombocythemia and polycythemia vera, carries a unique prognostic profile, with a markedly increased risk of thrombosis. This risk is the driving factor behind treatment strategies in these disorders. Clinical trials utilizing targeted therapies in thrombocytosis are ongoing with new therapeutic targets waiting to be explored. This paper will outline the mechanisms underlying thrombocytosis, the diagnostic evaluation of thrombocytosis, complications of thrombocytosis with a special focus on thrombotic risk as well as treatment options for clonal processes leading to thrombocytosis, including essential thrombocythemia and polycythemia vera.

## 1. Diagnostic Evaluation of Thrombocytosis

The threshold for clinically significant thrombocytosis is variable from patient to patient, and the exact definition of thrombocytosis also varies in the literature, although a platelet count of ≥450 × 10^9^/L is a generally accepted value [1]. A cohort study evaluating 10,000 Italian patients found a platelet count greater than 409 × 10^9^/L for women and 381 × 10^9^/L for men represented the 99th percentile in this population [2]. In this cohort, 99 patients (0.99%) had a platelet count greater than 400 × 10^9^/L on first measurement, with only 8 of these 99 exhibiting persistent thrombocytosis on re-evaluation 8 months later, reinforcing the importance of re-evaluation for persistence of thrombocytosis. Thrombocytosis has a multitude of potential etiologies and thus evaluation of a patient with thrombocytosis requires careful consideration of patient history, comorbid conditions, other hematologic parameters, and past platelet counts. In general, causes of thrombocytosis can be described as spurious, reactive, or clonal in nature (Table 1) [3]. 

A number of population studies have examined the degree of thrombocytosis as well as the frequency of various etiologies of thrombocytosis when it occurs. Reactive causes are by far the most common etiology of thrombocytosis in these population studies, comprising 88–97% of cases in adults in two large case series [4, 5] and 100% of pediatric cases in a single case series [6]. Extreme thrombocytosis, defined as a platelet count >1,000 × 10^9^/L is quite rare, as only 2–5.8% of patients demonstrate this degree of thrombocytosis upon presentation [4–6]. Although often thought to be more common in clonal processes, extreme thrombocytosis can also be due to reactive causes, with 82% of cases of extreme thrombocytosis in one series being reactive in nature [7]. 

### 1.1. Spurious Thrombocytosis

Spurious thrombocytosis is an extremely rare cause of apparent thrombocytosis, although it is likely underrecognized and characterized as reactive thrombocytosis in many cases as it often occurs along with processes associated with reactive thrombocytosis. Spurious thrombocytosis is characterized by the presence of nonplatelet structures in the peripheral blood which are counted as platelets by the automated counters used in modern complete blood counts. A variety of such structures, including needle-like cryoglobulin crystals [8], cytoplasmic fragments of circulating leukemic cells [9], bacteria [10], and red blood cell microvesicles following massive burn injury [11] are examples of the wide variety of structures that can mimic platelets when analyzed by automated cell counters. Peripheral blood smear evaluation is a simple method to confirm the veracity of a diagnosis of thrombocytosis and should be a part of every evaluation for a cause of thrombocytosis.

### 1.2. Reactive Thrombocytosis

**Figure 1:**
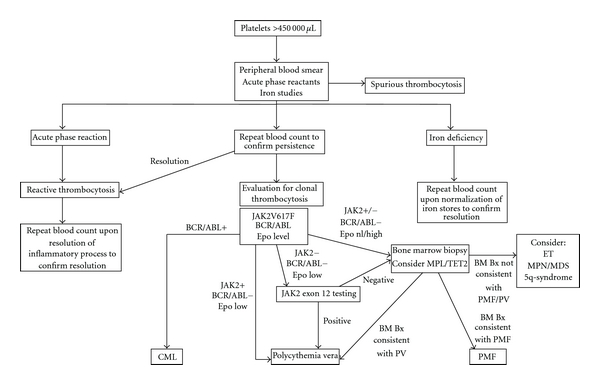
Diagnostic algorithm (Adapted from Harrison et al. and Tefferi et al.). BM Bx: bone marrow biopsy; CML: chronic myelogenous leukemia; Epo: serum erythropoietin; ET: essential thrombocythemia; MDS: myelodysplastic syndrome; PMF: primary myelofibrosis; PV: polycythemia vera.

Once the diagnosis of thrombocytosis is confirmed by peripheral blood smear review, the diagnostic evaluation turns to determining whether the process is reactive or clonal in nature (Figure 1). An important initial step in this determination is familiarity with the underlying causes of reactive thrombocytosis (Table 1). In adults, infection (typically acute), tissue damage, chronic inflammatory disorders, and malignancy are the most common causes of reactive thrombocytosis, with one or more of these processes present in >75% of cases of reactive thrombocytosis [4, 5]. In children, the most common causes of reactive thrombocytosis are similar, although hemolytic anemias, especially thalassemia, were a relatively common etiology in at least one Middle Eastern study [6]. A thorough history and physical examination should allow for the exclusion of multiple of the most common causes of reactive thrombocytosis.

The presence of a potential cause of reactive thrombocytosis does not rule out a concomitant clonal process, especially in persistent thrombocytosis. Much work has been done in an effort to come up with affordable, reliable, and rapid laboratory evaluation which can distinguish clonal processes from reactive thrombocytosis. The basis of many of these proposed evaluations is based on the mechanisms of reactive thrombocytosis.

Thrombocytopoiesis occurs in the setting of a complex cytokine milieu. Thrombopoietin (TPO) is the primary regulator of platelet production [12, 13], but many other cytokines such as IL-1 [14–16], IL-4 [17], IL-6 [14–22], IL-11 [23], and TNF [15] play important roles in thrombocytopoiesis. Many of these same cytokines also play a critical role in the body's response to inflammatory conditions [24, 25]. Evaluation of patients with reactive and clonal thrombocytosis has consistently demonstrated that circulating levels of multiple cytokines, most notably IL-6 [14, 17, 19, 20, 22, 25, 26] are elevated in patients with reactive thrombocytosis but not in those with clonal thrombocytosis or normal controls. Evaluation of circulating thrombopoietin levels as a discriminant between reactive and clonal processes has proved less informative, as results have not been consistent [18, 21, 22, 25–28]. One difficulty in utilizing circulating cytokine levels as a diagnostic tool lies in the finding that the rise in cytokine levels seems to precede the clinical finding of thrombocytosis, with levels returning to normal or near normal by the time thrombocytosis occurs [18, 21]. This fact, along with the difficulty in bringing such tests to clinical use, has led to a search for surrogate markers which may correlate with elevated cytokine levels, especially IL-6. Many other markers of the acute phase reaction, including C-reactive protein (CRP) [14, 19], ferritin [14], and erythrocyte sedimentation rate (ESR) [14], are also significantly elevated in patients with reactive as opposed to clonal thrombocytosis. 

Tefferi et al. [19] showed a correlation between IL-6 and CRP levels in a study of 91 patients with thrombocytosis, regardless of etiology. 76% of patients in this study with reactive thrombocytosis had an elevated CRP (>1.0 mg/dL), compared to 10% of patient with clonal thrombocytosis. Thus, measurement of CRP and other acute phase reactants can serve as easily obtained surrogates for measurement of cytokines important in thrombocytopoiesis and should be a part of any evaluation where reactive thrombocytosis is suspected. These surrogates are neither sensitive nor specific enough to allow them to play a definitive diagnostic role, but elevated acute phase reactants can support a diagnosis of reactive thrombocytosis in the right clinical setting. Other methods evaluated as ways to delineate reactive from clonal thrombocytosis include platelet indices [29], platelet function testing [30], and gene expression profiling [31]. 

Iron deficiency anemia is a common cause of reactive thrombocytosis [32], and evaluation of ferritin and iron studies should be a part of the evaluation of every patient with suspected reactive thrombocytosis. The pathophysiology of reactive thrombocytosis in iron deficiency anemia remains incompletely understood. Akan et al. demonstrated that levels of a number of cytokines typically elevated in reactive thrombocytosis (IL-6, IL-11, and TPO) were not elevated in patients with iron deficiency and thrombocytosis when compared to iron-deficient patients with normal platelet counts [33]. The levels of these cytokines did not change with iron therapy and resolution of thrombocytosis, indicating they likely do not play a major role in iron-deficiency-related thrombocytosis. The only hematopoietic cytokine found to be significantly elevated in this cohort was erythropoietin (EPO), which was elevated in patients with and without thrombocytosis. 

The role EPO plays in iron-deficiency-related thrombocytosis has garnered much interest, as administration of human recombinant EPO (rh-EPO) results in thrombocytosis of varying degree and duration in healthy controls [34] as well as patients with chronic kidney disease [35]. The physiology underlying this finding is controversial, as rh-EPO administration often leads to iron deficiency [36], leading to difficulty in determining whether elevated or exogenous EPO is a cause of thrombocytosis or simply a surrogate for iron deficiency. Studies, however, have consistently shown an increase in platelet count within 5 days of initiation of rh-EPO therapy [34, 35] regardless of serum ferritin levels [35], indicating a probable role for EPO in increasing platelet count independent of its effect of iron stores. It has been suggested that homology between the receptor for EPO (EPO-R) and TPO (MPL) may underlie this EPO-induced platelet rise [37], but *in vitro* studies have shown that EPO does not interact directly with MPL [38] and more likely plays a synergistic role along with TPO in stimulating platelet production [39, 40]. 

As this discussion demonstrates, the pathophysiology of reactive thrombocytosis is varied and complex. Even with all the diagnostic tools currently available, the diagnosis ultimately remains a clinical one based on laboratory findings, determination of a likely underlying cause, and, when possible, improvement with treatment of the underlying cause.

### 1.3. Clonal Thrombocytosis

**Figure 2:**
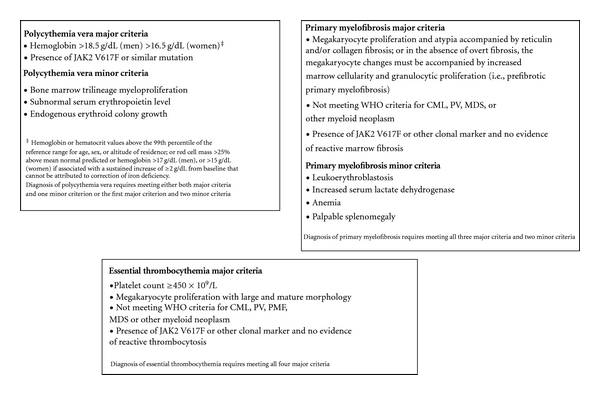
2008 WHO diagnostic criteria for PV, ET, and PMF. CML: chronic myelogenous leukemia; ET: essential thrombocythemia; MDS: myelodysplastic syndrome; PMF: primary myelofibrosis; PV: polycythemia vera.

Once a reactive thrombocytosis is excluded and thrombocytosis is persistent, the diagnostic evaluation should turn to distinguishing between the various causes of clonal thrombocytosis (Figure 1). The “classic” myeloproliferative neoplasms (MPNs), comprised of essential thrombocythemia (ET), chronic myeloid leukemia (CML), polycythemia vera (PV), and primary myelofibrosis (PMF) [41] are the most common clonal processes associated with thrombocytosis. This group of diseases is characterized by clonal expansion of a particular lineage of mature and/or maturing myeloid cells that arise from a hematopoietic stem cell. The 2008 World Health Organization (WHO) classification of Haematopoietic and Lymphoid Tissues [42] provides criteria for the diagnosis of all four “classic” MPNs, which are summarized in Figure 2. 

CML is characterized by dysregulated clonal expansion of all cells along the granulocytic maturation pathway. The etiologic abnormality and cornerstone for diagnosis of CML is the “Philadelphia chromosome”—a balanced translocation between chromosomes 9 and 22 leading to fusion of the *BCR *and *ABL1* genes and BCR-ABL1 fusion protein [43]. This translocation and resultant fusion protein is found in all patients with CML and can be detected by cytogenetics, fluorescence in situ hybridization (FISH), or reverse-transcriptase polymerase chain reaction (RT-PCR) [42]. Approximately 50% of patients with CML will present with thrombocytosis [44], usually in concert with a marked leukocytosis composed of cells in all phases of granulocytic maturation. However, a small number of patients will present with only mild or absent leukocytosis and thrombocytosis as the only hematologic abnormality [44], making evaluation for *BCR-ABL1* an essential component of any evaluation of clonal thrombocytosis, given the implications for management and prognosis a diagnosis of CML carries.

Discovery of *BCR-ABL1* fusion in CML served as evidence of clonality in this disease and led to a vigorous search for clonal molecular markers in the “Philadelphia chromosome negative” (Ph-) MPNs. This search has led to the discovery of multiple mutations crucial to our understanding of these disorders. The first of these discoveries occurred in 2005, when multiple independent groups described an acquired point mutation in exon 14 of the Janus kinase 2 (*JAK2*) gene resulting in a valine to phenylalanine substitution at codon 617 (*JAK2V617F*) [45–49]. JAK2 is a cytoplasmic tyrosine kinase with an important role in signal transduction in response to hematopoietic growth factors [50–52]. The JAK2V617F protein has been demonstrated to have constitutive activity independent of growth factor stimulation [45, 46, 48, 49], a likely explanation of the previously described autonomous hematopoietic colony formation [45–48, 53] and hypersensitivity to growth factors on *in vitro* testing of cells from patients with multiple MPNs [45, 47, 48, 54]. *JAK2V617F* is present in *≈*95% of patients with PV [45, 55], and *≈*40–60% with ET [45, 46, 55] and PMF [45–47].

The exact mechanism of how JAK2V617F alters cell proliferation remains to be elucidated, although multiple components of JAK2 function have been described and correlated with clinical features seen in the Ph- MPNs. JAK2V617F lies in the JAK2 pseudokinase (JH2) domain, which is an autoinhibitor of basal kinase function [56, 57]. Mutations within this domain lead to loss of autoinhibition and constitutive kinase activity [46, 58]. One of the predominant functions of JAK2 is the binding to and stabilization of homodimeric type I cytokine receptors, which have no kinase activity without this interaction. The receptors for erythropoietin (EPO-R), thrombopoietin (MPL), and other hematopoietic growth factors are homodimeric type I cytokine receptors [58–60]. Thus, constitutively active JAK2V617F, when paired with a homodimeric cytokine receptor, can lead to constitutive activity of that receptor independent of its ligand, leading to activation of the JAK-STAT and PI3K pathways [46, 58, 61], which are involved in cell proliferation and apoptosis, respectively. This interaction between JAK2 and homodimeric cytokine receptors may also be one reason for the phenotypic variation amongst patients with JAK2V617F, as the nature and frequency of these coreceptors on cells carrying JAK2V617F may impact the phenotypic expression of disease. 

Another potential underlying cause of the phenotypic diversity seen in *JAK2V617F* positive MPNs lies in the concept of allele burden. In general, *JAK2V617F* positive patients with ET have higher hemoglobin levels, lower platelet counts, and higher leukocyte counts than those without the mutation [62, 63]. Among those with *JAK2V617F*, a large spectrum exists as to the percentage of cells actually carrying the mutation, and this variation in allele burden has been correlated with differences in clinical phenotype. Patients with ET have the lowest allele burden, those with PV and PMF an intermediate one, and those with secondary myelofibrosis the highest burden [63–65]. Similarly, patients with PV are more likely to be homozygous for *JAK2V617F*, as 32% of patients were homozygous in one large case series; this is compared to only 2% of ET patients in the same series [66]. The role this variation in allele burden plays in other clinical manifestations of disease remains unclear.

Many unknowns remain as to the role of *JAK2V617F* mutations in the MPNs, especially given the wide phenotypic variation mentioned above, the fact that* JAK2V617F* is found rarely in other myeloid disorders [67, 68], and evidence that *JAK2V617F* may be a late occurrence in these disease, following a different, as yet unknown, causative mutation [69–71].

Given these unanswered questions regarding *JAK2V617F*, much effort has focused on identifying different mutations that may play a role in the Ph- MPNs. Multiple different mutations in exon 12 of the *JAK2* gene have been described and are detected in the majority of PV patients who lack the V617F mutation [72–74]. Although less well described, these mutations are also felt to interfere with the autoinhibitory function of JAK2 [75]. Multiple activating mutations in exon 10 of the *MPL* gene, which codes for the TPO receptor, have also been described [76, 77]. The prevalence of *MPL* mutations is approximately 5–7% of patients with PMF and 1–4% of ET patients [78, 79]; these mutations have not been described in patients with PV. *MPL *mutations have been detected concomitantly with *JAK2V617F*, although the percentage of Ph- MPN patients harboring a *MPL* mutation is higher in those without a JAK2 mutation [78]. Patients with *MPL* mutations do have a slightly different clinical phenotype, as they typically have lower hemoglobin levels and higher platelet counts than those without *MPL *mutations [80].

Another gene of interest in the MPNs is *TET2*, a putative tumor suppressor gene located on chromosome 4q24. Mutations of *TET2* have been detected in a variety of myeloid disorders, including Ph- MPNs. Approximately 13% of Ph- MPN patients harbor a *TET2* mutation [81], and it appears to be more common in patients >60 years old [82]. Clonal analysis also suggests that *TET2 *mutations can occur prior to or following *JAK2* mutation in patients where the two mutations exist concomitantly, indicating that *TET2* mutation is not a prerequisite for *JAK2* mutation [83]. Although the role of testing for novel mutations such as *MPL* and *TET2* as part of the diagnostic evaluation for the Ph- MPNs is less clear than that of *JAK2* evaluation, testing for these mutations has been shown to increase sensitivity when utilizing the WHO diagnostic criteria [84].

Even as the utility of molecular data in the diagnosis of Ph- MPNs increases, the diversity of disease present in the face of these mutations has required the diagnosis of the Ph- MPNs to remain primarily a clinical one, based on the 2008 WHO guidelines [42], summarized in Figure 2. Diagnosis of PV is based on the demonstration of increased red blood cell volume, depressed serum erythropoietin levels, and the presence of a clonal marker (*JAK2 *mutation) [85]. Thrombocytosis is also present in *≈*50% of cases of PV [86], and the presence of thrombocytosis in the absence of fever or infection is a minor criterion in the Polycythemia Vera Study Group diagnostic criteria for PV [86]. Although thrombocytosis is not a part of the WHO diagnostic criteria for PV, its presence, especially in the setting of evidence of elevated red cell volume, is entirely consistent with the diagnosis. Thrombocytosis can also be the only hematologic manifestation of PV, as the expected erythrocytosis can be masked by volume expansion or concomitant iron deficiency [87–89]. Diagnosis of PMF is based on the presence of typical findings on bone marrow biopsy, most notably reticulin fibrosis and megakaryocytic proliferation. Thrombocytosis is present in *≈*30% of PMF patients at diagnosis, with this number decreasing as the disease progresses and splenomegaly often leads to thrombocytopenia [90]. Diagnosis of essential thrombocythemia is essentially one of exclusion when no other diagnosis can be made in the setting of persistent clonal thrombocytosis.

## 2. Thrombotic Complications

Reactive thrombocytosis is generally felt thought to be a self-limited process which resolves with resolution of the underlying disorder when possible. The risk of thrombotic complications with reactive thrombocytosis is felt to be low, as 1.6% of patients with reactive thrombocytosis had thrombotic complications in one large case series [4]. All of these thrombotic events were venous in location and occurred in patients with other risk factors (postoperative setting or underlying malignancy). Even in cases of extreme reactive thrombocytosis, the risk of thrombotic complication is relatively low (4–6%) [91, 92], although reactive thrombocytosis has been shown to be an independent risk factor for thrombosis in patients otherwise at high risk for thrombosis, including trauma patients [93] and following coronary artery bypass grafting [94]. Patients in these settings have many other concurrent prothrombotic risk factors which likely contribute as much if not more to thrombotic risk than the presence of reactive thrombocytosis.

In clonal thrombocytosis, especially in ET and PV, thrombotic complications are a major cause of morbidity and mortality and the primary factor in determining treatment strategy. Although thrombosis can be an issue in other causes of clonal thrombocytosis, it is most common and most thoroughly investigated in PV and ET, and thus these diseases will be the focus of discussion. 

### 2.1. Microvascular Thrombotic Disease

Many patients with clonal thrombocytosis will experience vasomotor symptoms including headache, syncope, chest pain, erythromelalgia, acrocyanosis, and visual changes. These symptoms are due to microvascular inflammation, platelet aggregation and arteriolar microthrombi formation [95, 96] and are more common in ET than PV, with some vasomotor symptoms present in 29–40% of ET patients at presentation [97–99]. The frequency of erythromelalgia, the most common vasomotor complication of PV and ET, is not directly correlated with higher platelet counts, and in fact, vasomotor symptoms are essentially never present in reactive thrombocytosis. These facts underlie the important role that qualitative platelet abnormalities [96] and increased thromboxane-induced platelet activation [100] play in causing vasomotor symptoms, as erythromelalgia has been described in patients with relatively normal platelet counts in a multitude of nonhematologic conditions [101]. The lack of specificity of these symptoms makes estimating prevalence difficult, but the typical prompt response of ET-related vasomotor symptoms to aspirin can be a useful diagnostic as well as therapeutic intervention.

### 2.2. Macrovascular Thrombotic Disease

The rate of macrovascular thrombotic complications at diagnosis ranges 11–25% in ET and 12–39% in PV [102–106], with arterial thrombosis comprising the majority of events. The cerebrovascular circulation, either in the form of stroke or transient ischemic attack, is the most common site of arterial thrombotic disease, followed by the coronary arteries and peripheral vasculature [102, 105, 107, 108]. The arterial predominance of thrombotic events is more marked in ET than in PV, where up to *≈*40% of thrombotic events are venous in nature [102, 105, 109]. Of special significance are venous thromboses in unusual locations such as the splanchnic veins and cerebral sinuses, as >50% of venous thrombotic events in PV and ET occur in these locations [110, 111]; this is especially true in younger patients [112]. Given the frequency of thrombotic events in these unusual locations, strong consideration should be given to evaluation for occult Ph- MPN in any patient presenting with splanchnic or cerebral sinus thrombosis. Case series have reported that 23–51% of patients suffering from splanchnic thrombosis without any other risk factors can be diagnosed with an underlying Ph- MPN at the time of thrombosis [113, 114], and JAK2V617F mutations have been demonstrated in a number of such patients, many of which only met full criteria for a Ph- MPN later in their course [115, 116].

The risk for thrombosis does not stop with diagnosis, and large case series have demonstrated a significant risk of thrombotic events following diagnosis even in patients treated with cytoreductive and antiplatelet therapy. Wolanskyj et al. compiled a historic cohort of 322 patients with ET with 70% having >10 years of followup [106]. In this cohort, 26% of patients presented with evidence of thrombosis; the cumulative probability of thrombosis at 5, 10, and 20 years was 32%, 42%, and 52%, respectively. 82% of these patients received cytoreductive therapy and 62% received aspirin as part of their treatment regimen. Similarly, in a large series of 1213 Italian patients with ET [105] 34% of patients either presented with thrombosis (20%) or had a history of thrombosis prior to presentation (14%). After a median of 5 years of followup, an additional 19% of patients had suffered a thrombotic event, with a cumulative incidence of thrombosis of 53%.

### 2.3. Risk Factors for Thrombosis

Age is a significant risk factor for thrombosis in the general population [117], and multiple different epidemiologic studies have shown increasing risk for thrombosis in PV and ET with increasing age [102, 105–107]. Another consistently demonstrated risk factor for thrombosis in patients with PV and ET is a prior thrombotic event [98, 102, 105–107]. The combination of age >65 and a prior thrombotic event is associated with a markedly increased risk of thrombosis in PV, with a thrombosis rate in the European Collaboration on Low-Dose Aspirin in Polycythemia Vera (ECLAP) trial of 10.9 events/100 persons/year as compared to 2.5 events/100 persons/year in those without either risk factor [102].

Another more recently described independent risk factor for thrombosis in PV and ET is leukocytosis [106, 118–120]. The precise mechanisms behind this association are not entirely clear, although it is likely that qualitative leukocyte abnormalities play as large a role as quantitative increases in these diseases. The relative importance of qualitative leukocyte abnormalities is also supported by the fact that patients with CML, who typically have the highest leukocyte counts of all the MPNs, have a much lower rate of thrombosis than any of the Ph- MPNs [121]. Activated granulocytes are known to play an important role in platelet activation and endothelial injury [104], and patients with ET and PV have evidence of increased granulocyte activation as compared to normal controls [122]; an association between *JAK2V617F* and constitutive activation of granulocytes has also been suggested [64]. Another feature of this increased risk which speaks to a qualitative component is the degree of leukocytosis which confers an excess risk. Multiple series have shown that a leukocyte count of >8.7 × 10^9^/L, well within the normal range, is associated with excess thrombotic risk in both PV and ET [118, 120], with ROC analysis in one study showing a leukocyte count of 9.4 × 10^9^/L having the best sensitivity and specificity for demarcating high-and low-risk patients [118]. The implications of this association in regards to treatment of these disorders remain controversial, although it may provide a rationale for the efficacy of cytoreductive therapy and a leukocyte count of <10 × 10^9^/L has been integrated into recently revised European LeukemiaNet response criteria for PV and ET [123]. 

In contrast to the increased risk in leukocytosis, an increased risk of thrombotic events with increasing platelet count has not been consistently demonstrated. Multiple studies have failed to show a direct correlation between increasing platelet count and thrombotic risk [91, 107, 124], although *≈*80% of thrombotic events occur in those with a platelet count >600 × 10^9^/L [125]. Cytoreductive therapy leading to decreased platelet counts has been shown to decrease microcirculatory vasomotor symptoms and thrombosis [95, 125, 126], but it is unclear if this is directly due to decreases in the platelet count or to other effects of cytoreductive therapy such as a decrease in leukocyte count. Likely more important than the absolute number of platelets are the multiple qualitative abnormalities which have been noted in PV and ET, including decreased response to adenosine diphosphate and epinephrine [103, 127] altered glycoprotein receptors [127, 128], and the excess thromboxane production noted in the Ph- MPNs, leading to increased platelet activation [100, 129].

The role of erythrocytosis as an independent risk factor is similarly complex. Multiple studies have shown that increasing hematocrit is associated with increasing blood viscosity and thrombotic risk [130], with the cerebral circulation being especially vulnerable [131]. Increasing viscosity leads to a displacement of platelets to the periphery of arterial blood flow under high shear stress conditions [132], leading to greater interaction between platelets as well as platelets and the underlying endothelium. Many patients with PV also have an elevated number of platelets with the above-mentioned qualitative abnormalities, and bringing these platelets in closer contact with each other and the vessel wall is one likely reason for the increase in arterial thrombotic risk in these patients. These functional platelet abnormalities, as well as qualitative red blood cell (RBC) abnormalities including increased RBC adhesion to endothelial cells [133], likely play an important role in thrombosis in PV and ET, as animal models have failed to show hyperviscosity alone as a significant risk factor for thrombosis [134].

Evaluation of the role of traditional cardiovascular risk factors to thrombotic risk in PV and ET has led to conflicting results, with some studies suggesting increased risk with hypercholesterolemia [107] and smoking [135] while others have shown no increased risk with any known cardiovascular risk factors [106, 124]. 

The discovery of the *JAK2V617F* mutation has led to extensive evaluation to determine whether those carrying this mutation have a different disease phenotype than those who are *JAK2* wild type, and this evaluation has included examining the risk of thrombosis in both groups. A theoretical basis for an increased risk of thrombosis in PV and ET lies in the fact that the increased RBC-endothelial adhesion mentioned above is felt to be mediated by a *JAK2V617F*-mediated mechanism [133]. Thus far, the implications of *JAK2V617F* mutation on thrombosis risk are unclear, with multiple retrospective studies indicating an increased risk of thrombosis in ET [136–138], while others have failed to show such an association [118, 139]. A recent meta-analysis of 21 studies in ET patients suggested an increased risk of increased risk of thrombosis (OR 1.92, 95% CI 1.45–2.53) in those with *JAK2V617F* mutations [140]; the increased risk was for both venous and arterial thromboses. Other groups have examined increasing *JAK2* allele burden as a risk factor for thrombosis and a potential reason for the conflicting results regarding the impact of *JAK2* mutations on thrombotic risk. Analysis of 639 patients with ET and 323 with PV by Vannucchi et al. reported an increased risk of thrombosis (both arterial and venous) in the small percentage of ET patients who were homozygous for *JAK2V617F* (hazard ratio 3.97, 95% confidence interval [CI] 1.34–11.7) compared with JAK2 wild-type patients [66]. No such difference was present in patients with PV and other studies have not demonstrated this increased risk in ET patients [118].

The impact of *MPL* mutations on thrombotic risk has been less well evaluated and is difficult given the small number of patients with these mutations and its frequent cooccurrence with JAK2 mutations. In the small number of studies evaluating thrombotic risk in these patients, *MPL* mutation does not appear to be an independent risk factor for thrombosis [78, 80].

## 3. Bleeding Risk

A paradoxical risk of bleeding has been noted in patients with thrombocytosis, especially extreme thrombocytosis [91, 98], and bleeding in this setting is usually mucocutaneous in nature. This excess risk is likely multifactorial; in clonal thrombocytosis, platelet function abnormalities no doubt play a major role. Another potential cause of bleeding regardless of the cause of thrombocytosis is acquired von Willebrand's syndrome (AVWS) due to increased adsorption of large von Willebrand factor (vWF) multimers [141, 142] by the abnormally high number of circulating platelets. Testing for AVWS can be undertaken in patients with thrombocytosis and bleeding to evaluate for a decrease in large vWF multimers, and in patients with extreme thrombocytosis, evaluation for AVWS can be incorporated into the risk stratification of otherwise low-risk patients prior to the initiation of antiplatelet therapy.

## 4. Treatment of Thrombocytosis

### 4.1. Reactive Thrombocytosis

Reactive thrombocytosis, as mentioned above, is felt to be self-limited with little excess associated thrombotic risk. Because of this lack of thrombotic risk as well as a theoretical risk of paradoxical bleeding, no antiplatelet therapy is recommended, even for extreme thrombocytosis. The occurrence of thrombosis in patients felt to have reactive thrombocytosis may be reason to undertake evaluation for a concomitant clonal thrombocytosis, especially with splanchnic or cerebral thrombosis. 

In patients who present with extreme thrombocytosis of unknown etiology and evidence of active bleeding or critical thrombosis, plateletpheresis can provide a rapid reduction in platelet count while the diagnostic evaluation is undertaken [143, 144]. In patients where a clonal cause of thrombocytosis is known or suspected, cytoreductive therapy (discussed below) can also be initiated at high doses for additional rapid platelet-lowering effect [145].

### 4.2. Clonal Thrombocytosis

**Figure 3:**
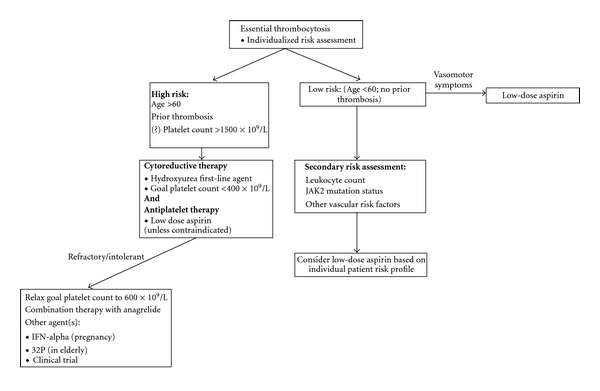
Treatment algorithm for ET.

**Figure 4:**
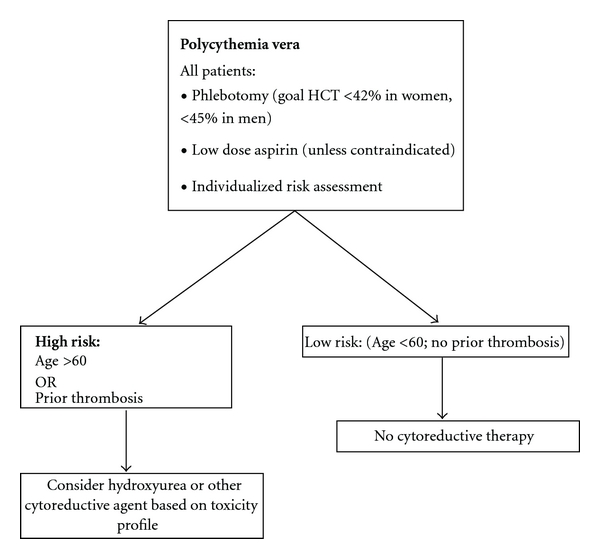
Treatment algorithm for PV.

Treatment strategies in PV and ET are focused on reducing the risk of thrombotic events in those at risk for them, as thrombosis is the most common complication leading to morbidity and mortality in these disorders. Treatments to this end fall into essentially two categories: cytoreductive therapy with an aim to decrease circulating platelet count (as well as hematocrit in PV) and antiplatelet therapy, usually in the form of aspirin. Given these treatment approaches, much work has been done in an effort to risk-stratify patients to determine which patients will benefit from either or both of the above classes of therapy. Risk assessment and subsequent therapy based on risk of thrombosis has led to a variety of treatment strategies based on risk (Figures 3 and 4).

### 4.3. Essential Thrombocytosis

In ET, multiple retrospective studies have consistently described age >60 and a prior thrombotic event as risk factors for thrombosis [98, 106, 107]. Patients meeting either of these criteria are generally considered to be in a “high risk” group and felt thought to benefit from both antiplatelet and cytoreductive therapy. This approach is supported by multiple randomized clinical trials. The first, by Cortelazzo et al. [126] prospectively evaluated 114 patients with ET who met the above high-risk criteria. Patients were randomized to receive hydroxyurea (HU), an antimetabolite which primarily acts in S phase and is effective in reducing platelet counts in ET, versus no cytoreductive therapy. HU dose was titrated to maintain a platelet count <600 × 10^9^/L and after a median of 27 months of followup, 3.6% of patients receiving HU had suffered a thrombotic event, compared to 24% in the control group, a statistically significant difference. It should be noted that 69% of patients were on some form of antiplatelet therapy (aspirin or ticlodipine), and use of these agents was similar in the two treatment groups. Whereas this trial demonstrated the benefit of cytoreductive therapy, the Medical Research Council primary thrombocythemia-1 (PT-1) trial [146] aimed to determine the best method of cytoreduction, comparing the use of HU with anagrelide. Anagrelide is an orally active quinazolinone derivative which was initially developed as an inhibitor of platelet aggregation but has also been shown to reduce platelet count with little effect on other hematopoietic cell lines through blockade of megakaryocyte differentiation and proliferation [147]. In the PT-1 trial, a total of 809 patients who met high-risk criteria (defined as any of the following: age >60 years, prior thrombosis, hypertension or platelet count >1,000 × 10^9^/L) were randomized to receive low-dose aspirin plus either HU or anagrelide titrated to maintain a platelet count <400 × 10^9^/L. Patients randomized to HU + aspirin had a significantly lower rate of arterial thrombosis, major hemorrhage, and transformation to myelofibrosis, but significantly higher risk of venous thrombosis. There were no differences in control of platelet count in the two groups, and the overall risk of thrombosis after a median of 39 months of followup was 7.6% in the HU group compared to 10.1% in the anagrelide group.

These results have led to widespread adoption of HU + aspirin as first-line therapy in patients with high-risk ET. Anagrelide + aspirin does appear to have benefit in reducing thrombotic events in patients in ET, if one compares thrombotic rates in the PT-1 trial with that of the study by Cortelazzo et al. [126] and other historic controls. Thus, anagrelide continues to have an important role in the treatment of ET, especially when toxicities such as cytopenias or cutaneous lesions limit the use of HU. There is growing evidence that combination therapy with HU and anagrelide can be effective in treating patients either refractory to or intolerant of large doses of HU [148]. Compared to treatment with anagrelide alone, this combined approach allows for continuation of reduced dose HU, which may allow for continued leukocyte reduction, which likely contributes at least a portion of the protective benefit of HU treatment.

Goals of treatment in high-risk ET are primarily defined in terms of reduction in platelet count, with a goal of <400 × 10^9^/L being a common target given the findings of the PT-1 trial [146]. Patients in the study by Cortelazzo et al. [126] had a targeted platelet count of <600 × 10^9^/L, indicating that a slightly relaxed platelet goal may still allow for benefit in cytoreductive therapy in those unable to maintain a platelet count of <400 × 10^9^/L. The recently published European LeukemiaNet response criteria for ET [123] include many other factors, with complete response defined as (1) platelet count <400 × 10^9^/L, (2) no disease-related symptoms, (3) normal spleen size on imaging, and (4) white blood cell count <10 × 10^9^/L. Partial response is defined as platelet count <600 × 10^9^/L or a decrease of >50% from baseline. The utility of these criteria in decision-making regarding treatment strategies in ET is yet to be determined. 

Growing evidence supports the concept that patients with ET who do not meet high-risk criteria do well with less aggressive therapy. Rugerri et al. [149] prospectively followed 65 patients <60 years old with no thrombosis history and a platelet count <1500 × 10^9^/L, and the thrombosis rate in this cohort (1.91/100 patient-years) was not significantly different than that of age and sex-matched controls (1.5/100 patient-years). Aspirin was used only in the event of microvascular symptoms; less than 25% of patients in both arms received aspirin during the median 4.1 years of followup. 

Antiplatelet therapy, most commonly aspirin, also plays an important role in the treatment of ET, and its efficacy in the treatment of microvascular complications has been well established [95, 96]. Its role in the prevention of macrovascular complications is less clear. Retrospective evaluation suggests antiplatelet therapy may reduce thrombotic complications in a high-risk population [124, 150], but randomized data is lacking. Further support for the use of antiplatelet therapy in ET has been extrapolated from the ECLAP trial, which demonstrated a significant reduction in vascular events in low and intermediate risk PV patients treated with aspirin as compared to placebo [151].

The role of antiplatelet therapy in a low-risk ET population was recently examined in a retrospective evaluation of 300 Spanish patients by Alvarez-Larran et al. [152]. Rates of thrombosis were not significantly different in those treated with antiplatelet therapy and those followed with observation; subgroup analysis did demonstrate that patients harboring the *JAK2V617F* mutation did have a significantly increased rate of venous, but not arterial thrombosis. Patients with cardiovascular risk factors who were observed without antiplatelet therapy also had a higher rate of arterial thrombosis compared to those receiving aspirin. Patients with a platelet count of >1000 × 10^9^/L treated with antiplatelet therapy had a significantly increased risk of bleeding. These data reinforce the importance of a thorough, individualized approach to risk assessment in ET patients prior to determining a treatment strategy.

### 4.4. Polycythemia Vera

The foundation of therapy in polycythemia vera is therapeutic phlebotomy to maintain a hematocrit <45% in men and <42% in women (Figure 4). This approach is based on the phlebotomy strategy utilized in multiple randomized PVSG studies that demonstrated phlebotomy alone was associated with equivalent or improved survival when compared to phlebotomy plus additional agents such as busulfan, 32P, and chlorambucil [153, 154]. Addition of these agents was consistently associated with increased risk of late hematologic complications, especially acute myeloid leukemia. Treatment with phlebotomy alone, however, was associated with a relatively high risk of thrombosis in these studies, especially early in the course of therapy, with thrombosis rates of 23% in the first two years. 

In an effort to address this early thrombotic risk, a subsequent Phase II PVSG trial including 51 patients added HU to phlebotomy with a goal hematocrit <50% and platelet count <600 × 10^9^/L. The thrombotic rate seen in the first 2 years of therapy was 9%, significantly lower than the 23% noted in the earlier PVSG trial [155]. The risk of leukemic transformation in this cohort was 6% and not significantly different than that seen in the phlebotomy only arm of earlier PVSG studies, but the leukemogenic risk of HU is still hotly debated [156–158]. No phase III studies comparing HU + phlebotomy with phlebotomy alone in PV have been performed, but the results of this Phase II study have led to the common use of HU in addition to phlebotomy in those considered high risk for thrombosis (Age >60 and/or prior thrombosis) [159]. 

The use of antiplatelet therapy in PV was initially thought to be associated with poorer outcomes and increased bleeding risk based on an early PVSG study [160]. This protocol, however, utilized a total dose of 900 mg of aspirin daily along with dipyridamole, another antiplatelet agent. Subsequent studies have demonstrated the efficacy and safety of lower doses of aspirin therapy in PV. A Gruppo Italiano Studio Policitemia Vera study [161] randomized 112 PV patients to receive either aspirin (40 mg/day) or placebo. The low dose aspirin was well tolerated, led to a significant decrease in serum thromboxane B_2_ levels, and was not associated with an increased risk of bleeding. The ECLAP trial [151] randomized 518 patients who had no other indication for antiplatelet therapy to low-dose aspirin or placebo and demonstrated significant reduction in the risk of nonfatal myocardial infarction, nonfatal stroke, pulmonary embolism, deep venous thrombosis, and death. Bleeding risk was not significantly different in the two treatment groups. These data have led to recommendations for use of low-dose aspirin in all patients with PV, regardless of risk, except in the face of contraindication. 

Other agents, such as interferon alpha [162, 163] and, in Europe, pipobroman [164, 165] have also been used in the treatment of both ET and PV. The use of these therapies is generally limited to those who have failed or are intolerant of more standard therapy; interferon alpha is generally considered the therapy of choice in pregnant patients requiring cytoreductive therapy given its low teratogenicity [166]. A variety of JAK2 inhibitors have been evaluated in preclinical studies in the Ph- MPNs and are currently progressing through clinical trials [167, 168]; the majority of patients enrolled in JAK2 inhibitor trials to this point have had PMF. Results have been promising, and further trial results and evaluation of new potential therapeutic agents may lead to a paradigm shift in the treatment of all the MPNs, including ET and PV.

## 5. Conclusion

Thrombocytosis exists in the setting of a variety of clinical situations and can have widely diverse underlying etiologies. Clonal thrombocytosis is often due to one of the Ph- MPNs, and in this setting, the risk of thrombosis is great and should be the primary factor guiding treatment strategy. The mechanisms underlying this increased thrombotic risk are not yet fully understood, and the role of risk factors such as JAK2 status and leukocytosis has yet to be conclusively established. Integration of these and other risk factors into a risk-based treatment decision model should allow for better patient selection for cytoreductive therapy. The ongoing discovery and description of new molecular abnormalities in these disorders should allow for advances in both diagnosis and treatment of clonal thrombocytosis.

## Figures and Tables

**Table 1:** Causes of thrombocytosis (adapted from Harrison et al.).

Clonal	Reactive	Spurious
Essential thrombocythemia	Infection	Microspherocytes
Polycythemia vera	Inflammation	Cryoglobulinemia
Primary myelofibrosis	Tissue damage	Neoplastic cell fragments
Myelodysplasia with del (5q)	Hyposplenism	Schistocytes
Refractory anemia with ringed	Post-operative	Bacteria
sideroblasts associated with		
marked thrombocytosis (RARS-T)		
Chronic myeloid leukemia	Iron deficiency	
Chronic myelomonocytic leukemia	Malignancy	
Atypical chronic myeloid leukemia	Hemolysis	
MDS/MPN-U	Drug effect	
POEMS syndrome	“Rebound” following myelosuppression	
Familial thrombocytosis		
